# Geminiviruses and Plant Hosts: A Closer Examination of the Molecular Arms Race

**DOI:** 10.3390/v9090256

**Published:** 2017-09-15

**Authors:** Shunmugiah V. Ramesh, Pranav P. Sahu, Manoj Prasad, Shelly Praveen, Hanu R. Pappu

**Affiliations:** 1 ICAR-Indian Institute of Soybean Research, Indian Council of Agricultural Research, Indore 452001, India; ramesh.sv@icar.gov.in; 2 Department of Plant Pathology, Washington State University, Pullman, WA 99163, USA; 3 National Institute of Plant Genome Research, Aruna Asaf Ali Marg, New Delhi110067, India; prnv_pankaj@yahoo.co.in (P.P.S.); manoj_prasad@nipgr.ac.in (M.P.); 4 Division of Plant Pathology, Advanced Centre for Plant Virology, ICAR-Indian Agricultural Research Institute (IARI), New Delhi 110012, India; shellypraveen@hotmail.com

**Keywords:** geminivirus, plant-virus interactions, cellular reprogramming, small non-coding RNAs, viral suppressors

## Abstract

Geminiviruses are plant-infecting viruses characterized by a single-stranded DNA (ssDNA) genome. Geminivirus-derived proteins are multifunctional and effective regulators in modulating the host cellular processes resulting in successful infection. Virus-host interactions result in changes in host gene expression patterns, reprogram plant signaling controls, disrupt central cellular metabolic pathways, impair plant’s defense system, and effectively evade RNA silencing response leading to host susceptibility. This review summarizes what is known about the cellular processes in the continuing tug of war between geminiviruses and their plant hosts at the molecular level. In addition, implications for engineered resistance to geminivirus infection in the context of a greater understanding of the molecular processes are also discussed. Finally, the prospect of employing geminivirus-based vectors in plant genome engineering and the emergence of powerful genome editing tools to confer geminivirus resistance are highlighted to complete the perspective on geminivirus-plant molecular interactions.

## 1. Introduction

Geminiviruses are plant-infecting viruses with a monopartite or bipartite single-stranded DNA (ssDNA) encapsidated in twinned icosahedral particles. The *Geminiviridae* comprises nine different genera, viz., *Becurtovirus*, *Begomovirus*, *Capulovirus*, *Curtovirus*, *Eragrovirus*, *Grablovirus*, *Mastrevirus*, *Topucovirus*, and *Turncurtovirus*. The delineating criteria include the vector, host range, genome organization and sequence relationships [1,2].The individual icosahedron encapsidates ssDNA which is ~2.7–3.0kb and, upon infection, double stranded replicative form is generated inside the plant nucleus [3,4,5]. The genome of geminiviruses comprises either two DNA components (bipartite: DNA A and DNA B) [6] or a single DNA component (monopartite) [7,8].

For bipartite geminiviruses, the DNAA component encodes proteins associated with viral DNA replication, encapsidation, vector transmission and viral suppressors of RNA silencing (VSRs), whereas DNAB encodes proteins involved in intercellular and intracellular movement of virus particles [9,10]. Monopartite viruses comprise a single DNA component as the genome which is homologous to DNAA of bipartite viruses hence, the movement functions are provided by the coat protein gene, or V2 ORF [11]. However, in many instances, monopartite begomoviruses are associated with additional ssDNA components referred to as alpha- or beta-satellites. The satellite DNA components are partly or entirely dependent on the helper virus for their replication, movement and encapsidation functions. In some instances, the proteins encoded by satellite DNA components associated with monopartite viruses act as VSRs [12]. The alpha-satellites (DNA α) are characterized by self-replicating ssDNA ((~1375 nucleotides (nt)) half the size of the single DNA component of begomoviruses, and thus are dependent on the helper virus for movement, encapsidation and vector transmission [13]. Alpha-satellites encode replication initiator protein and share features of origin of replication with *Nanoviridae* [13]. Monopartite begomoviruses are also associated with small circular ssDNA components (of size ~1.3–1.4 kb) called beta-satellites (DNA β) [14]. Unlike the alpha-satellites, beta-satellites are pathogenicity determinants that depend completely on their helper virus for replication, encapsidation and vector transmission functions [15,16].

The genomic components of geminiviruses evolve rapidly and genetic recombination among the various genomic components is a major factor driving their evolution [17,18]. Interestingly, genetic recombination tends to preserve the protein–protein or protein–DNA interactions that play a major role in evolution and diversity [18]. Recombination between the helper virus and alpha-satellite components has also been documented in field conditions [19]. Similarly, recombination between helper virus, *Tomato yellow leaf curl China virus* (TYLCCNV) and beta-satellites resulted in the development of a recombinant virus with expanded host range [20]. A novel begomovirus genome-derived recombinant satellite molecule (RecSat), which is a recombinant of alpha- and beta-satellite DNA components of three parents (Tomato yellow leaf curl China beta satellite, *Ageratum* yellow vein China beta-satellite and Tobacco curly shoot alpha-satellite), was found [21]. Although no apparent effect of RecSat on the symptom development in *Nicotiana benthamiana* was observed, it helped in increasing the helper virus accumulation, suggesting some biological role reminiscent of defective interfering DNAs.

Geminiviruses are the subject of interest in molecular virology owing to their relatively small genome, and their ability to master and reprogram host cellular processes to their advantage [17]. The geminivirus genome is dependent on host DNA and RNA polymerases for its replication and transcription. The ssDNA genome is replicated into double-stranded DNA (dsDNA) form, which is found to be associated with host histone proteins as mini-chromosomes inside the nucleus [10,22,23]. Furthermore, geminiviruses cause significant economic losses in food, feed and fiber crops affecting food and nutritional security worldwide. For instance, severe losses in cotton in Asia [24], and cassava [25] and maize in Africa [26] are attributed to geminivirus infection. Legumes in India are infected with begomoviruses that cause annual yield losses estimated at $300 million [27]. Tomato leaf curl viruses (ToLCVs) cause annual yield losses of $140 million in Florida, USA and continue to be a constraint to tomato production worldwide [28,29].This review summarizes our current knowledge of the molecular arms race between geminiviruses and their host plants and its implications for developing engineered, virus resistant crops, with particular emphasis on the geminivirus-induced RNA silencing mechanisms and the counter silencing measures.

## 2. Geminiviruses and RNA Silencing

**Figure 1:**
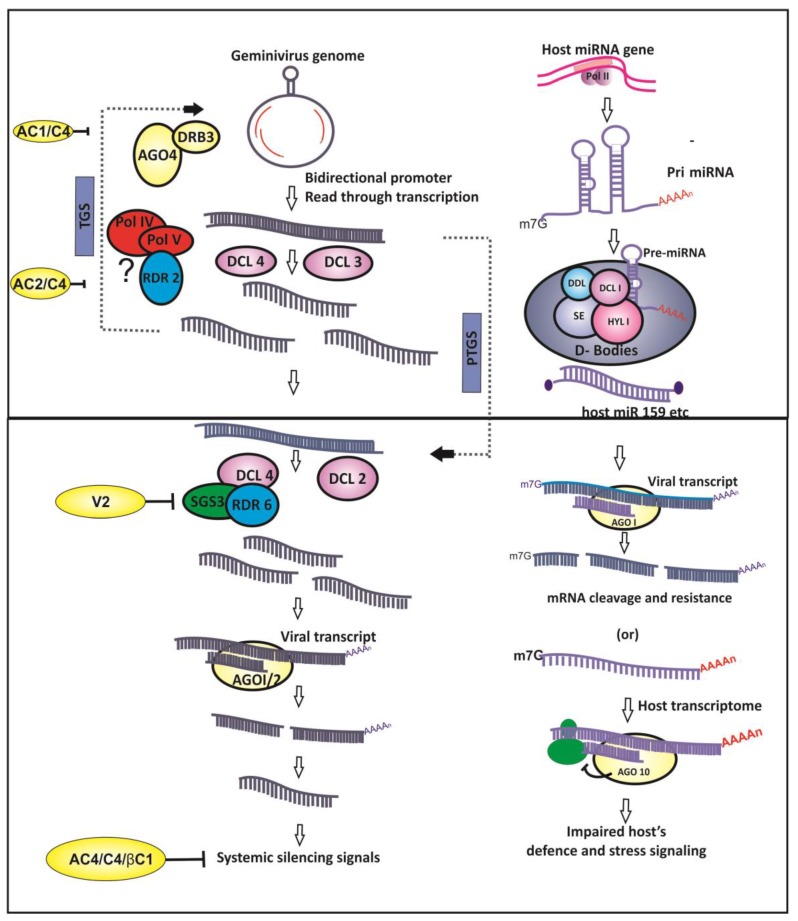
Geminivirus and host interactions at the small RNA interface. The host RNA silencing machinery targets viral transcripts via small interfering RNAs (siRNAs) generated from the viral genome and through host-derived microRNAs (miRNAs). Viral suppressors of RNA silencing (VSRs) counter host RNA silencing based defense, and impaired defense is also manifested due to interference in miRNA-mediated gene regulatory network.

Geminiviruses are inducers of RNA silencing owing to the bidirectional transcription of their genomes [10], aberrant read-through transcription [30], and the overlapping ORFs which results in the production of double-stranded RNA (dsRNA)—a key instigator of the RNA silencing pathways (Figure 1).

Small RNA (sRNA)-based gene silencing, a sequence-dependent process, results in homologous mRNA degradation or translational repression at post-transcriptional level, referred to as post-transcriptional gene silencing (PTGS). In addition, sRNAs also direct methylation of cognate DNA at transcriptional level in RNA-dependent DNA methylation (RdDM), resulting in epigenetic suppression of gene expression. This mechanism of repressing the transcription of the target DNA owing to its sRNA-mediated methylation results in transcriptional gene silencing (TGS).Taken together, both PTGS and TGS mechanisms of RNA silencing are functional as antiviral defense during geminivirus infection in plants and control the expression of viral genes [31,32,33] (Figure 1).

Upon geminivirus infection, RNA silencing of target viral mRNA is directed by viral genome derived small interfering RNAs (vsiRNAs). vsiRNAs originate from the dsRNA precursors by the activity of RNAse III type endonucleases called as Dicer-like (DCL) enzymes. Another important component of RNA silencing comprises Argonaute (AGO) proteins that are an integral part of slicers that make up the RNA-induced silencing complex (RISC).The helicase activity of AGO is required for unwinding of the siRNA duplexes and single-stranded guide siRNA recruitment into RISC [34].The mature siRNAs in RISC are provided stability by the HUA enhancer-1(HEN1)-mediated methylation [35]. In PTGS, the RISC present in the cytoplasm cleaves the target mRNA which exhibits sequence complementarity to the recruited siRNA (Figure 1). Though the exact triggering mechanism for PTGS during geminivirus infection is unknown, it is speculated that the host RNA-dependent RNA polymerases (RdRPs) act on the transcripts of opposite polarity extending them to generate long dsRNAs—the actual trigger for PTGS. Moreover, the abundant, early viral transcripts could also act as a template for host RdRP-mediated generation of dsRNAs, subsequently leading to the production of siRNAs.

Transcriptional suppression of geminivirus genome has been known to occur in plant protoplasts and the inhibition of virus replication is mediated by DNA methylation [36].Later, it was proven that virus entry into transgenic plants leads to selective epigenetic suppression of homologous regulatory elements in viral genomes [37]. Viral genome silencing is not only associated with cytosine methylation but also with histone modification (H3K9) [38]. However, conclusive evidence for viral genome methylation as a mode of plant’s defense strategy came from plant methylation mutants that displayed greater susceptibility to viral infection. Mutants of methylation pathway such as chromatin remodelers (*ddm1*), *AGO4* and RNA polymerase IV subunit (*nrpd2a*) showed enhanced vulnerability to *Beet curly top virus* (BCTV) and *Cabbage leaf curl virus* (CaLCuV) infections [39]. Likewise, hyper-susceptibility was observed with plant mutants lacking non-CG methyl transferases (*drm1*/*drm2* and *cmt3*), H3K9 methyl transferases (*kyp2*/*suvh4*) and adenosine kinase mutants (*adk1* and *adk2*).Although mutants belonging to CG methyl transferases are detrimental, heterozygous mutants were more susceptible to virus infections and thus emphasizing the role of non-CG methylation pathway in plant’s defense mechanism [39]. Furthermore, bisulfite sequencing of viral DNA genomes revealed that disease severity was correlated with deficient host methylation patterns. Moreover, contrasting histone modifications, with reference to the state of gene activation such as, methylations (H3K9me2) and acetylations (at K9 and/or K14) were observed at the intergenic region (IR) of the viral genomes during CaLCuV infection. These findings suggest the co-existence of both active and repressive genomic marks in infected plants [39].

The connection between the plant’s antiviral defense mechanisms and its methylation process is established once again in studies involving recovery processes, wherein new tissues of infected plants would emerge virus-free or with low virus titer. The recovery from viral infection was correlated to the plant’s ability to methylate the virus genome [40,41]. Moreover, *ago4* plant mutants could not recover from BCTV infection, suggesting methylation of viral genomic components is a basic defense pathway in plants [39]. Similarly, the methylation-mediated recovery was observed in Cucurbit leaf crumple virus-infected watermelon. In an otherwise susceptible or poor recovery zucchini genotype, the dsRNA was shown to generate siRNAs targeting viral intergenic region (IR) resulted in recovery phenomenon [42]. Similarly, recovery from Pepper golden mosaic virus infection is correlated with methylation of the viral genome [43]. Inside the nucleus, 24 nt siRNAs are generated by DCL3 processing of the RdRP2-generateddsRNA precursor. These 24 nt siRNAs are stabilized by the methylation activity of HEN1 and are recruited into RNA-induced transcriptional silencing (RITS) complex with the help of AGO4, AGO6, or AGO9. The RITS complex, comprising of the 24 nt-long siRNAs is implicated in RNA-directed DNA methylation [44].

## 3. Geminivirus and Small Non-Coding RNAs

Geminivirus infection triggers the process of RNA silencing in the host [31]. The effector molecules of RNA silencing pathway are small RNAs that regulate gene expression in a sequence-dependent manner. Small RNAs (sRNAs) not only play a major role in fundamental cellular processes such as defense against viral invasion [45] but also in the temporal and spatial regulation of gene expression [46], maintenance of chromatin status [47], transposon mobility control, and in histone methylation [48]. The main classes of sRNAs are small interfering RNAs (siRNAs) and microRNAs (miRNAs) [49]. In plants, these sRNAs act in RNA silencing pathways and regulate the expression of both plant and viral derived genes.

Unlike RNA silencing in animals that encode one kind of Dicer, plants encode multiple DCL enzymes and the activity of each dicer generates siRNAs of characteristic lengths [31].The siRNAs of varied lengths, such as 21, 24 and 22 nt, are produced by the activity of DCL2, DCL3 and DCL4, respectively, during geminivirus infections [31,50,51] (Figure 1). Generally, 21–22 nt siRNAs target viral coding regions, whereas 24 nt siRNAs were found to be targeting IR of the viral genome [31]. Geminivirus-plant interactions with respect to sRNAs is characterized by the production of abundant 24 nt siRNAs, suggesting TGS mode of virus gene suppression is the predominant antiviral mechanism [31].The entire geminivirus genome is subjected to vsiRNA-mediated methylation; however, methylation hot-spots vary depending upon the host-virus system. The siRNAs were primarily mapped to the IR or promoter regions of the virus genome; however, generation of vsiRNAs from the coding region is not uncommon [31,50,52,53]. Introduction of in vitro methylated viral DNA as replicons into the plant system caused about 20-fold decline in the virus population [36], proving that the host-induced virus genome methylation is a major antiviral defense mechanism even though progeny viral genomes have been found to be non-methylated. Furthermore, enhanced viral DNA methylation occurs when the genome is a linear, heterogeneous DNA molecule [54]. These findings divulge that geminiviruses are subject to host-directed TGS mechanism due to the effect of the24 nt vsiRNAs. On the other hand, DCL4 and DCL2 generated 21 and 22 nt siRNAs prime the AGO1 and AGO2, respectively, leading to the slicing of the target virus transcript in the cytoplasm [31,34].

Besides the silencing effects of the primary siRNAs, RNA silencing pathway is characterized by the production of secondary siRNAs due to the amplification of the silencing signals. The secondary siRNAs are formed due to the activity of host-derived RNA polymerases IV and V and RNA dependent RNA polymerase 2 (RDR2) in the TGS pathway leading to the DCL3-mediated production of 24 nt siRNAs [55,56]. Unlike RNA viruses, wherein RDR2 is known to play a major role in the production of secondary siRNAs, direct evidence for the involvement of RDR2 is yet to be elucidated in geminivirus infection [32]. Mutations involving RDR6 cause a little increase in viral DNA, suggesting that secondary siRNAs might play a vital role in the defense against viral infection [56,57]. It was also speculated that the role of VSRs in suppressing the methyl cycle might be an important cause for the inactivation ofRDR2 in geminivirus infection [58]. Recently, it was found that DRB 3 (dsRNA binding protein 3) interacts with DCL3 and AGO4 and is considered to be involved in RNA-directed DNA methylation (RdDM) during geminivirus infection [59].

MicroRNAs are the class of 21-nt-length small non-coding RNAs (ncRNAs) that function as crucial effector molecules of PTGS in plants. Although the antiviral nature of host-derived miRNAs is abundant in animal–virus interactions, few instances of plant miRNA-mediated antiviral activities are also documented (Figure 1). Microarray analysis of tomato plants agro-infected with *Tomato leaf curl New Delhi virus* (ToLCNDV) showed the down-regulation of conserved miRNA families such asmiR319 and miR172 [60]. However, the miRNAs involved in plant developmental processes were up-regulated leading to the suppression of corresponding endogenous targets during another begomovirus infection [61]. Computational prediction of miRNA-based antiviral resistance revealed that several tomato-derived passenger miRNA strands (miRNA*) showed propensity for binding to ToLCNDV-associated genomes. In silico prediction [62] and micro-array or quantitative real time PCR (qRT-PCR)-based expression analysis of host-derived miRNAs in tomato [60] and soybean [63] showed that these conserved miRNAs display a greater inclination to target viral genomes. Differential regulation of in silico- predicted antiviral miRNAs appeared to be targeting tomato leaf curl virus in resistant and susceptible genotypes of tomato [64]. In addition, the plausible role for the putative antiviral miRNAs and their target genes in the development of symptoms and resistance mechanism was postulated [63,64]. Furthermore, the abundance of plant conserved miRNAs has led to the speculation that they are chiefly conserved for their function in antiviral resistance [65].The antiviral activity of miRNAs is further corroborated by the activity of geminivirus-derived VSR, AC4 protein, that binds sRNAs including host miRNAs and debilitates the miRNA mediated host defense mechanism [66]. On the other hand, the miRNAs derived from the genomes of animal-infecting viruses target the host defense mechanism. The plant virus-derived miRNAs have not been reported yet because of the absence of the phenomenon of latent infection with phytopathogenic viruses. In addition, the majority of plant viruses are RNA viruses and, do not enter the host nuclei. Considering these facts, the genome of geminiviruses could be examined for its miRNA coding potential because geminiviruses enter the plant nucleus. Interestingly, the expression of viral derived miRNAs has been demonstrated during the infection of African cassava mosaic virus and East African cassava mosaic virus—Uganda [67]. Hence, the proof of miRNA coding potential and functional miRNAs from the genomes of a geminivirus raises questions as to who determines the outcome of the host-geminivirus interactions in the sRNA interface [67].

## 4. Geminivirus and Counter-RNA Silencing

RNA silencing mechanism is primarily an immune response to the invading pathogens such as viruses. As a counter-defense, viruses have evolved proteins called viral suppressors of RNA silencing (VSRs) (Figure 1). Initial reports of geminivirus encoded VSRs showed that the expression of *African cassava mosaic virus*(ACMV) and *Tomato yellow leaf curl virus* (TYLCV) encoded AC2 proteins could reverse the RNA silencing already set in plants [68,69]. Further, it was observed that the AC2 suppressor activity is consistent with its active nuclear localization signal (NLS) and DNA binding domains. It suggests that geminiviral VSRs influence RNA silencing in a manner different from the RNA viruses as the latter predominantly binds sRNAs to inhibit RNA silencing [69,70]. Later, the necessity for NLS and activation domain in silencing suppression was demonstrated for legume begomovirus (*Mungbean yellow mosaic virus*) (MYMV) encoded AC2 protein, which also functions as a transactivator of virus promoters. Thus, both the promoter transactivation and suppression of RNA silencing functions of AC2 encoded by Mungbean yellow mosaic virus-Vigna were interconnected [71]. In addition, AC2 encoded by ACMV did not reveal any sRNA (miRNAs and siRNAs) binding ability differing significantly from VSRs encoded by RNA viruses, for example, tomato bushy stunt virusP19 [66]. However, the silencing suppression is correlated with the transactivation of host transcript(s) by *Mungbean yellow mosaic India virus* (MYMIV) encoded VSR (AC2). It is noteworthy that amongst the host transcripts that were up-regulated by AC2, WEL1 (Werner exonuclease-like 1) was shown to suppress RNA silencing in *N. benthamiana* [71]. Prior to this, homologous proteins AL2 and C2 encoded by Tomato golden mosaic virus (TGMV) and BCTV, respectively, were shown to interact with and inactivate host serine/threonine kinase (SNF) related kinase (SnRK1) and adenosine kinase (ADK) that are involved in the host methyl cycle maintenance [39,72,73]. ADK is an essential enzyme for maintenance of host methyl cycle and *S*-adenosyl methionine (SAM) dependent methylation, whereas SnRK1 is a key regulator of the host metabolism. The VSR-kinase interactions occur in the cytoplasm hence, could not account for the transactivation of host genes. These transcription-independent studies also demonstrated that silencing suppression could be achieved by truncated AL2 that lacked the activation domain. Inhibition of ADK by adenosine analogs and or dsRNA-mediated down-regulation also mimicked the effect of geminivirus protein inactivation of ADK [41].

The symptom recovery phenomenon during virus infection occurs in the absence of viral opposing factor. For instance, BCTV encoded L2 protein disrupts methylation pathway of plants, however, deletion of L2 protein helps in the recovery of the plants from virus induced symptoms [40,41]. In addition, C2/L2 encoded by curtovirus (Spinach curly top virus) lacks transcriptional activation domain, and hence, transcriptional activation is not observed [74]. Thus, geminivirus-derived suppressor protein AC2 and its positional homologs such as AL2/L2/C2 interfere with host methylation mediated suppression of viral gene expression. Suppressor proteins AL2 encoded by begomovirus (CaLCuV) and L2 encoded by curtovirus (BCTV) show differential suppressor activity depending on the vegetative or reproductive stage of the plants [75]. Furthermore, a novel suppression strategy employed by AL2 which is dependent neither on ADK inhibition nor on transcription activation has been reported [75].

Exploration of host factors interacting with Beet severe curly top virus (BSCTV) encoded C2 identified *S*-adenosyl methionine decarboxylase 1 (SAMDC1) as an interacting partner. Further, C2 improves in vivo stability of host SAMDC1 by arresting its ubiquitylation and proteasome-mediated degradation [76]. Interestingly, replication initiator protein has also been implicated in the suppression of TGS mechanism by altering the expression levels of host methyl transferases such as methyltransferase 1 (MET1), and chromomethylase 3 (CMT3) in a manner similar to that in animal DNA viruses [32].

Multiple geminivirus infections in plants cause synergism among the infecting viruses which is manifested in an increased severity of symptoms. Unraveling the molecular mechanism underlying the synergism, led to the discovery of ORF AC4 derived VSR [77]. AC4 protein coded by an embedded ORF within the AC1 ORF of ACMV-CM and Sri Lankan cassava mosaic virus (SLCMV) acts as a suppressor of PTGS. Enhanced viral synergism has been linked to the complementation effect of AC4 and AC2 genes encoded by African cassava mosaic virus—Cameroon strain (ACMV—(CM)) and East African cassava mosaic Cameroon virus (EACMCV), respectively [78]. Unlike AC2 that inactivates the host methyl cycle, AC4 interacts with single-stranded siRNAs and miRNAs but not with duplex siRNAs [66]. Thus, AC4 acts on the downstream step of the small RNA synthesis in the RNA silencing pathway. Structural examination of C4/AC4 proteins (pathogenicity factor and VSR in some cases) of several geminiviruses infecting a range of crops have shown a conserved, consensus *N*-myristoylation motif required for eliciting disease-like symptoms, membrane binding and pathogenicity. AC4 protein encoded by ACMV—[CM] is a silencing suppressor that directly interacts with host miRNA pathway. Reportedly, AC4 protein binds with single-stranded miRNA or siRNA and inhibits the miRNA-mediated negative regulation of gene expression, culminating in developmental defects in *Arabidopsis* [66]. Thus, viral suppressor of RNA silencing perturbs the host miRNA pathway, leading to alterations in the stress signaling pathways linked to the hormonal regulations and ultimately resulting in abnormal phenotypes. These findings suggest that interference with miRNA-directed processes might be a common feature of viral pathogenicity [76,79]. In addition, the involvement of DRB3 in miRNA-mediated gene repression and siRNA-mediated methylation suggests that the antiviral mechanism concertedly acts as a molecular network. Molecular characterization of VSRs of cotton leaf curl virus-beta-satellite complex (*Cotton leaf curl Multan virus* (CLCuMV) and Cotton leaf curl Multan β-satellite (CLCuMβ) revealed that AC4 encoded by CLCuMV binds both long and short RNAs with a preferential binding of dsRNA [80]. Thus, suppressor AC4 is a multi-functional protein that interferes with both upstream and downstream of siRNA duplex unwinding process followed by its incorporation into RISC. Further, AC4 interferes with RNA silencing pathway by sequestering both long dsRNA from DCL cleavage and siRNA from RISC incorporation [80].AC5 protein encoded by many bipartite begomoviruses function as VSR. Unlike AC2, AC5 encoded by MYMIV suppresses sense RNA-induced gene silencing but not RNA silencing triggered by dsRNA, suggesting that thus AC5 interferes with dsRNA production [81].

Monopartite viruses are constrained due to their smaller genome size (almost half the size of bipartite viruses and has only one DNA component) that encodes all the proteins necessary to complete the infection cycle. Monopartite TYLCCV encoded C2 protein, which is a homolog of AC2 or AL2 of bipartite viruses, functions as a VSR besides acting as a host-range determinant [71]. The cysteine residues in the putative zinc finger motif of C2 protein are indispensable for its anti-RNA silencing function [69]. BSCTV encoded VSR C2 inhibits the methyl cycle by attenuating the degradation of *S*-adenosyl methionine decarboxylase 1 (SAMDC1) [76]. However, in monopartite viruses TYLCV and TYLCCV, an unlikely candidate, V2 protein, was reported to suppress RNA silencing. Interestingly, it acts downstream of the dicer activity in the RNA silencing pathway at the level of amplification of the silencing signals [82,83]. TGS suppression activity of V2 encoded by TYLCV was established by reversal of the TGS-based GFP silencing in *N. benthamiana* line 16-TGS [84]. Transgenic expression of V2 leads to significant reduction in methylation of host genomic regions, suggesting its role in the suppression of TGS [83].

Viral suppressors such asV2 and AC4 act on the amplification step of RNA silencing hence prevent the spread of silencing signals. Aberrant RNA is processed into 21 nt siRNAs by the concerted action of host RDR6, suppressor of gene silencing3 (SGS3) and DCL4, however, V2 competitively inhibits the activity of SGS3 and suppresses the amplification of silencing signals [84]. Similarly, long distance movement of 24 nt siRNAs is counteracted by AC4 and beta-satellite encoded βC1 [80,83].

Not much is known about the mechanism of beta-satellites in suppressing RNA silencing even though beta-satellite encoded βC1 was found to bind ssDNA or dsDNA in a non-specific manner and localizes in the nucleus. Co-infection of TYLCCV and βC1 in *N. benthamiana* 16c plants (wherein GFP expression is silenced) reversed the silencing phenomenon suggesting that βC1 is a VSR [85]. Despite sharing DNA binding and nuclear localization properties with AC2 or L2, the βC1 expression is associated with developmental abnormalities. Hence, it is speculated that βC1 might act on the sRNA pathway overlapping with the host miRNA gene regulatory mechanism [86]. Similarly, VSRs of other beta-satellites such as Bhendi yellow vein mosaic beta-satellite [87] and Tomato leaf curl Java beta-satellite associated with *Ageratum* yellow vein virus—Indonesia(AYVV—ID) [88] were characterized. Likewise, Cotton leaf curl virus-beta-satellite complex (Cotton leaf curl Multan virus(CLCuMV) and Cotton leaf curl Multan beta-satellite (CLCuMB)) revealed as many as four VSRs (V2, C2, C4, and βC1 proteins) [80]. Similar modes of suppression of RNA silencing by C4 and βC1 were observed as both the suppressors showed propensity for binding long dsRNAs, suggesting their involvement in the interference at DCL cleavage of dsRNA and sequestration of siRNAs [80].

Investigations into the suppressor functions of βC1 encoded by three beta-satellites (Tomato leaf curl Bangalore beta-satellite (ToLCBB), Cotton leaf curl Multan beta-satellite (CLCuMB) and Luffa leaf distortion beta-satellite (LuLDB)) associated with TYLCV revealed that all the three suppressors were able to reverse the silencing of GFP expression in *N. tabacum* cv. Xanthi. However, ToLCBB-derived βC1 acted on the mRNA degradation to siRNA formation step, whereas the other two suppressors inhibited the silencing maintenance activity [89]. Recently, the βC1 encoded by DNA satellite associated with TYLCCV showed that it interacts with *N. benthamiana* calmodulin like protein (Nb-rgs CAM) causing its up-regulation. Thus, this study revealed that VSR activates endogenous suppressors of RNA silencing (ESRs). This interaction represses host RDR6 and ultimately impedes the production of secondary siRNAs thus proving the indispensability of RDR6in geminivirus-host interactions [90]. Besides, AC2 and its positional homologs, begomovirus beta-satellite encoded βC1 alters host methylation-mediated virus defense pathway by inhibiting *S*-adenosyl homocysteine hydrolase [12].

Other geminiviruses such as mastreviruses encode VSRs [91]. The Rep protein encoded by *Wheat dwarf virus* (WDV) inhibits RNA silencing and spread of silencing signals [91]. Electrophoretic mobility shift assays showed that WDV-encoded Rep protein binds 21–24 nt single-stranded and duplex siRNAs [91]. In addition, the Rep coded by geminiviral alpha-satellites was reported to function as the suppressors of RNA silencing. The alpha-satellite associated with *Cotton leaf curl Rajasthan virus* (CLCuRaV) beta-satellite complex maintains the viral rep protein mediated suppressor activity in young cotton tissues [92]. It accounts for the selective advantage of association of alpha-satellites with begomovirus beta-satellite disease complex.

Begomoviruses have evolved multiple strategies that include numerous viral suppressor proteins to counteract the host RNA silencing mechanism [80,82]. Multiple suppressors encoded by geminivirus warrant the quantification of relative strengths of the suppressor activity. In the Cotton leaf curl virus and beta-satellite complex, even though V2 is known to have the strongest suppressor activity [80], it cannot be construed as the major VSR. The actual suppressor activity in planta might vary as viral genes are differentially regulated in both time and space during the infection process. Furthermore, findings from the model plants such as *N. benthamiana* and *Arabidopsis* have to be confirmed in the natural hosts of the virus. The greatest challenge is in engineering geminivirus resistance in plants based on RNA silencing because of multiple suppressors acting at different stages of silencing process which might lead to the breakdown of small RNA-based resistance.

## 5. Geminiviruses and Host Cellular Reprogramming

The defense and counter-defense strategies between geminiviruses and plants have become an interesting area of research. Besides the VSRs that alter the host RNA silencing pathway, geminiviruses also encode proteins that modulate host cellular programming. Molecular-competition for survival involves an array of proteins from the host plants, whereas geminiviruses have only 5–7 proteins. In most cases, viruses are capable of overcoming the defense strategies of plants, due to the complex nature of viral proteins, which are designed to interact dynamically with diverse corresponding plant proteins.

### 5.1. Redirection of Host Gene Expression

**Figure 2:**
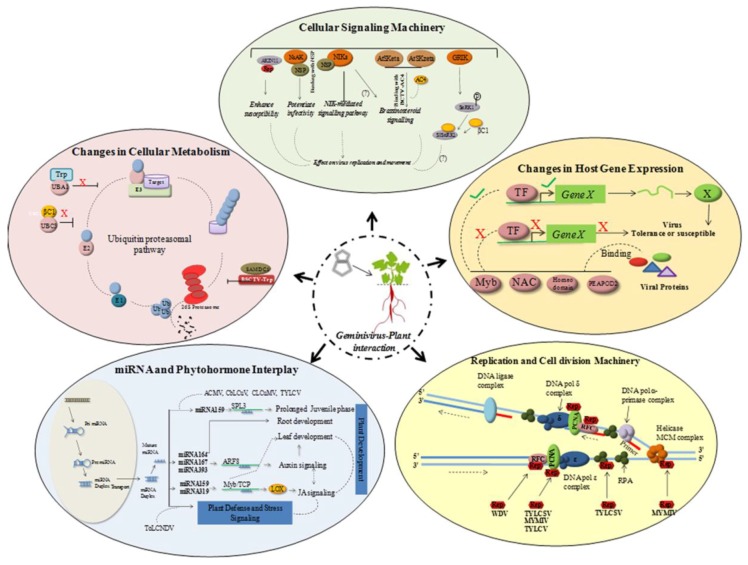
Geminivirus–plant host interactions. Geminivirus entry into plant host induces changes in gene expression patterns, cellular signaling process, replication, cell division, protein degradation and miRNA-phytohormone interplay.

The geminivirus genome encodes replication-associated protein (Rep/AC1/C1) only, and thus is dependent on the host replication machinery to amplify its genome. In order to overcome the inadequate host DNA replication factors in the differentiated cells, the virus redirects host gene expression to accumulate host DNA polymerases to assist the viral genome amplification [93]. Geminivirus proteins interact with host transcription machinery, DNA replication and cell division-related proteins, plant metabolic process proteins, defense-related proteins, and stress-related proteins [94] (Figure 2).

Rep plays a pivotal role in stimulating the replication of viral and plant DNA. It can bind to the subunits of host DNA polymerase complex [95,96,97,98], ssDNA binding proteins [99,100], and recombination/repair proteins to interfere in host DNA replication process (Figure 2). They also interact with host transcription factors responsible for regulating the host gene expression patterns. Diverse transcription factor families such as, NAC, Myb, homeodomain-leucine zipper and PEAPOD2 were shown to positively or negatively regulate viral infection in various plant-virus interactions [58,101] (Figure 2). CaLCuV infection in *Arabidopsis* revealed that geminivirus influences cell cycle genes differentially and causes progression into endocycle [102]. Geminivirus encoded proteins have been demonstrated to interact with host protein kinases implying their role in altering signal transduction pathways. In some cases, reduced activity of the host protein kinases is required (reduced activity of SNF1-related kinase (SnRK1) due to binding of viral protein) for effective viral infection cycle [103], whereas in other instances an increased activity of receptor-like kinases (RLKs) is essential for the infection process [104]. It is also evident that diverse host gene expression pathways including genes involved in systemic acquired resistance (SAR), WRKY TFs [105], cell cycle PCNA [106], are also altered following virus infection. Upregulation of defensin-like proteins, PR1 proteins and other LRR domain containing proteins, along with some transcription factors such as NAC or WRKY, have been associated with the induction of defense-related genes and innate immunity [107,108]. This suggested a trade off in the plant developmental processes during viral infection.

### 5.2. Host Hormonal Signaling

During infection, geminivirus penetrate, establish and move to the neighboring cells for further dissemination throughout the plant. Interactions of viral and cellular factors may not only contribute to facilitate these steps and help establish optimum susceptibility conditions, but may also indirectly affect host physiological processes. Although the exact nature of virus-induced symptom development with stress signaling responses is generally unknown, it appears to be tightly networked. Phytohormones play a very important role in controlling host physiological responses which get altered during viral infections; hence it is important to study hormonal regulation in the context of stress signaling pathways. Auxin biosynthesis and jasmonic acid (JA) signaling share many commonalities [109] and are interrelated by a plausible network (Figure 2; interrelation of auxin signaling and stress-responsive JA pathway). The JA biosynthetic gene *LOX2* (chloroplast encoded lipooxygenase2) is regulated by miR319/TCP activity [110]. In addition, mir319/TCP node has been found to be involved in the regulation of miR164/CUC node as a part of the auxin signaling network [111]. These cascades of responses are supposed to be regulating the transcriptional changes in the host during viral infection. Although the relationship between altered gene expressions during disease development is yet to be explored, many viral proteins known as pathogenicity/virulent factors are being supposedly involved in causing disease like symptoms [112]. They also disrupt cell signaling molecules to reduce host cellular defense activation. For instance, BCTV-C4 and cabbage leaf curl virus-NSP interact with LRR receptor like kinase in *N. benthamiana*, which is an important signaling molecule against pathogen defense [113,114].The cell-to-cell movement of geminivirus requires the assistance of host proteins such as nuclear acetyl transferase [100], importin α, synaptotagmin [115] and transport GTPase [116].

Viral infection also affects hormone biosynthesis. Several host genes associated with auxins/IAA, ethylene-responsive factors, cytokinin and gibberellins were found to be differentially regulated during ToLCNDV infection [108].

### 5.3. Altered Host Protein Degradation Pathways

Geminiviral proteins have also been reported to impede host protein degradation pathways to redirect protein metabolism in the plant cell. The majority of viral proteins were shown to interact with plant ubiquitination, SUMOylation and protease-mediated degradation machinery to alter plant developmental and defense processes [58,94]. TYLCSV-derived C2 has been shown to interfere with derubylation activity of the CSN (COP9 signalosome) complex thereby inhibiting the activity of SCF (for Skp1/Cullin1/F-box) complexes which are involved in host ubiquitylation pathway. In addition, the ultimate target of geminivirus-triggered inhibition of SCF complexes was found to repress the jasmonate signaling pathway [100]. Similarly, BSCTV-C2 impairs host-derived E3 ligase inhibiting SAMDC1 degradation which, in turn, leads to the inhibition of methylation-dependent viral gene silencing [76]. However, BSCTV-derived C4 induces E3 ligase and causes degradation of cell cycle regulators thereby affecting cell cycle related pathways. Inhibition of E2 enzyme by CLCuMV encoded βC1 causes decrease in global polyubiquitination that further leads to perturbation of developmental and hormonal signaling pathway [117]. However, the replication initiator protein (Rep) encoded by TYLCSV, ACMV, and TGMV, interacts with E1SUMO-conjugating enzyme so that the host SUMOylation is hampered [118]. Strikingly, most genes which are known to be influenced by the TYLCSV infection were found to be involved in post-translational modifications (PTMs) [119] which is not surprising since PTM is considered to be a rapid and effective way of responding to virus infection. Further, increasing evidences indicate that such PTMs are targets for viral proteins in order to alter host’s defense response because hosts manipulate the PTMs as a defense against virus infection. Thus, geminivirus proteins have functionally evolved the capability to usurp the host proteins involved in ubiquitination and thereby modulate the protein degradation system to its advantage. Recently, it has been demonstrated that the beta-satellite DNA molecule associated with geminiviruses induces host calmodulin-like protein (CAM), which is an endogenous RNA silencing suppressor. *N. benthamiana* derived calmodulin-like protein (NbCaM) was shown to interact with suppressor of gene silencing 3 (NbSGS3) and degrades it. This NbCaM mediated degradation of NbSGS3 during geminivirus infection is sensitive to autophagy inhibitors. Thus, autophagy also plays an important role in the suppression of RNA silencing pathway during geminivirus infection [120].

### 5.4. Impaired Cellular Metabolism 

Interference in and/or modification of host cellular metabolism helps the virus to multiply in the host by redirecting nutrients, and modifying the cell wall synthesis and structure [121]. Functional annotation of altered transcripts during tomato leaf curl infection in tomato suggests that viral infection results in the alteration of metabolism of carbohydrates, starch, sugars and amino acids. Up-regulation of phosphoenol pyruvate phosphate and acetyl Coenzyme A transcripts during infection suggests changes in this important biochemical network, central to cellular metabolism, which includes glycolysis and TCA cycle [108]. Capsid protein (CP) has also been shown to hinder glyoxylase and shikimate pathways. The role of stress-responsive proteins in the development of virus infection was also reported in various studies. Recently, interaction of TYLCSV-AC4 and TYLCSV-AV2 with wounding induced F14P1.1and dehydration-responsive RD21 [122],respectively, was reported in *N. benthamiana*. Global transcriptomic changes upon monopartite TYLCSV infection in tomato showed the up-regulation of genes involved in phytohormone metabolism, nucleic acid metabolism and ubiquitin-autophagy pathway, whereas the down-regulated genes were involved in primary metabolism, methylation-dependent chromatin silencing [123]. Interactions of curtovirus derived C4 and *Arabidopsis thaliana* SHAGGY-like protein kinases (AtSKs) were implicated in hyperplasia observed during BCTV infection [124].

## 6. Implications for Engineered Resistance

From the plant protection perspective, it is inevitable to consider that geminiviruses are master re-programmers of host cellular processes. Geminiviruses encode numerous VSRs [86]. On the hosts’ side, the diversity of geminivirus VSRs and their varied modes of action require an effective defense strategy. It is advisable to channelize the RNAi silencing-based genetic modification toward the down-regulation of geminivirus derived VSRs to target the focal pathogenicity determinant in virus infection cycle. Effective negative regulation of VSRs employing siRNAs [125,126], artificial miRNAs (amiRNAs) [127,128], and artificial transacting siRNAs [129] has resulted in considerable degree of virus resistance. However, widespread incidence of geminivirus infections under field conditions combined with multiple infections might lead to complementation or synergism effect among the unrelated viruses [77]. Hence, in such conditions, genetically engineered resistance based only on the RNA silencing phenomenon would be inadequate. As such, it is imperative to search for innate host genes that confer resistance or tolerance to virus infection. In this regard, the recent finding of geminivirus resistance genes *Ty*-1 and *Ty*-3 from *Solanum chilense* against TYLCV is encouraging [130]. The successful combination of natural resistance with RNA silencing against two geminiviral diseases, cassava brown streak disease (CBSD) and cassava mosaic disease (CMD) in cassava is worth considering in managing geminiviruses in legumes where the viruses seem to evolve through recombination and trans-replication [131].

The complex interactions between host cellular machinery and geminivirus-derived proteins also provide an opportunity to develop geminivirus resistant genotypes. From these interactions, it appears that the replication initiator protein is a suitable target for engineering durable resistance either by expressing siRNAs [125,126] or through transgenic expression of rep-binding peptide aptamers [132]. Recently, it was shown that *Nicotiana* plants employ salicylate- and ethylene-dependent extreme resistance (ER) as a counter-counter defense strategy against the infection of RNA virus, *Tomato bushy stunt virus* (TBSV) [133],where the P19 suppressor whose sRNA binding is imperative for the observed ER in *Nicotiana tabacum*. It is tempting to draw parallels with the geminivirus–host interactions where aVSR-AC4was implicated in counteracting the host defense by sRNAs binding. Hence, a search for the counter-counter defense strategy in geminivirus-plant interactions might result in finding a similar phenomenon of extreme resistance. One recent addition to genetic engineering approaches is the deployment of prokaryotic immunity system with a characteristic clustered regularly interspaced short palindromic repeats (CRISPRs)/CRISPR associated 9 (Cas9) protein that has emerged as a potent genome editing tool to confer geminivirus resistance. The approach allows targeted modification of viral genomic DNA with the transgenic expression of a single guide RNA (sgRNA) that provides specificity to Cas9 endonuclease. Emerging research proved that genome editing could potentially be harnessed to confer geminivirus resistance for *Bean yellow dwarf virus* (BeYDV) and Beet severe curly top virus (BSCTV) in *N. benthamiana* [134,135]. Furthermore, CRISPR/Cas9-mediated targeting of geminiviral genome’s intergenic, conserved region has a potential to target multiple viruses [136]. Ali et al. [137] showed that CRISPR/Cas9-mediated targeting of viral non-coding or intergenic regions was effective than targeting the viral coding region, as viral variants capable of replication and systemic infection were generated when the target region included the coding sequences.

## 7. Future Directions and Conclusions

Most plant-infecting viruses evolved to be RNA viruses, implying viruses with DNA genomes are at a disadvantage in the plant sub-cellular system. However, geminiviruses clearly make the case that they are an exception. It is important to identify the interface components of geminivirus-plant interactions which are essential for the establishment of successful infection. It will be a difficult task to disrupt host protein network and their functions without affecting normal plant development. However, the molecular insights gained to date are giving us only a brief idea of targeted host proteins involved in virus multiplication and symptom development. Global expression studies and proteome analysis in the infected cells would provide a more complete picture of the host proteins that are regularly subverted by the virus proteins to help the establishment of virus infection. Once available, it offers an opportunity to identify the pathways that are vulnerable to virus infection and to find ways to prevent the same.

Although various studies have correlated the RNA silencing with host tolerance against geminivirus infection [53,138,139,140], still many questions need to be answered; for instance, how these vsiRNAs move all over the plant tissues to signal/trigger the defense response. A better understanding about, how the plant effectively activates RNA silencing machinery with the help of host counterparts to facilitate movement could provide some clues regarding the antiviral immunity in plants. In addition, the exact origin of dsRNA from the geminivirus infection remains unclear. Overlapping ORFs is considered to be a prime reason for dsRNA formation, suggesting that sRNA profiling would yield siRNAs only from the overlapping region however that was not the case as small RNAs have been mapped to the entire genome. Although the direct role of host RDRs (RDR2 or RDR6) in the generation of dsRNA is yet to be identified in geminivirus infection, an indirect function for RDR6 in the generation of secondary siRNAs is encouraging [90]. In addition, the only host resistance genes for geminivirus infection identified to date, i.e., *Ty-1* and *Ty-3* [130], exhibit homology to host RDR class, which signifies the importance of secondary siRNAs in conferring antiviral resistance. However, elaboration and evidence are required before concluding that secondary siRNAs are, in fact, the effector molecules of RNA silencing-based resistance to geminiviruses. Thus, a greater understanding of the transcriptional and post-transcriptional RNA silencing that is triggered upon geminivirus infection is important in order to be able to devise effective control strategies. Decoding the molecular processes in the virus infection cycle using “omics” approaches such as next generation sequencing (NGS) and microarrays could identify the myriad of host protein factors that are targets of virus infection. The real challenge lies in applying the insights gained thus far in developing a stable crop genotype that has durable resistance to geminivirus infection.

Geminivirus genomes, owing to their relatively small size, offer an enormous opportunity for use in novel applications. A virus-induced gene silencing (VIGS) vector using TYLCV minimal genome and the viral amplicon (VA) vector with null AC2 were successful in silencing tomato PCNA gene, suggesting the utility of the geminivirus-based VIGS approach [141]. Prior to this, a deconstructed genome of *Bean yellow dwarf virus* (BeYDV)-based geminivirus vectors have been effectively used in the expression of heterologous proteins [142,143]. A recent potent addition to the utility of geminivirus based vector is its application in plant genome modification via gene targeting. It involves the introduction of precise double-stranded breaks using sequence specific nucleases (ZFN (zinc-finger nucleases) [144], TALENs (transcription activator-like effector nucleases) [145], and CAS (Clustered, regularly interspaced, short palindromic repeat (CRISPR)-associated protein system)). The improved knowledge of the molecular biology of geminiviruses enabled researchers to use BeYDV based geminiviral replicons (GVRs) for delivery of the larger nucleases such as TALENs and CAS system along with repair template for successful genome modification of plants [146]. The GVR-based gene targeting opens up a wide array of possibilities not only in genome editing of higher plants but also in studies involving functional genomics because of its high copy number, its applicability to a wide host range, compatibility with T-DNA based gene insertions, and the amenability for introduction of larger genes. Plant genome engineering based on GVRs help not only in incorporating a trait but also in engineering knock-outs that have a role to play in gene function studies and in the development of plants devoid of anti-nutritional factors. Geminivirus based vectors have been successfully utilized in rice to achieve over 19% increased frequency of DNA knock-in(KI) lines signifying the versatility of geminivirus genomes to achieve homology directed repair (HDR) [147]. Smaller genome size, increased cargo capacity, and multiple hosts are some of the attributes of geminiviruses that offer enormous opportunities in genome editing. On the other hand, CRISPR/Cas9 mediated genome editing tools have been employed to develop geminivirus resistant plants. Recent advancements in this field have identified targeting viral non-coding or intergenic region as a powerful approach to achieve durable resistance.

## Abbreviations

ADK	adenosine kinase
AGO	Argonaute
Cas9	CRISPR-associated 9
CRISPRs	Clustered regularly interspaced short palindromic repeats
DCL	Dicer-like enzyme
HEN	HUA Enhancer
miRNA	MicroRNA
NLS	Nuclear localization signal
PTGS	Post-transcriptional gene silencing
PTM	Post translational modification
RdDM	RNA-dependent DNA methylation
RdRP	RNA-dependent RNA polymerase
RecSat	Recombinant satellite molecule
Rep	Replication initiator protein
RISC	RNA-induced silencing complex
RITS	RNA-induced transcriptional silencing
SAM	S-adenosyl methionine
SAMDC	S-adenosyl methionine decarboxylase
siRNA	Small interfering RNA
sncRNAs	Small non-coding RNAs
SnRK	Serine/threonine kinase (SNF) related kinase
TGS	Transcriptional gene silencing
vsiRNA	Viral genome derived small interfering RNA
VSR	Viral suppressors of RNA silencing
